# Insulin-Independent and Dependent Glucose Transporters in Brain Mural Cells in CADASIL

**DOI:** 10.3389/fgene.2020.01022

**Published:** 2020-09-15

**Authors:** Mahmod Panahi, Patricia Rodriguez Rodriguez, Seyed-Mohammad Fereshtehnejad, Donia Arafa, Nenad Bogdanovic, Bengt Winblad, Angel Cedazo-Minguez, Juha Rinne, Taher Darreh-Shori, Yoshiki Hase, Raj N. Kalaria, Matti Viitanen, Homira Behbahani

**Affiliations:** ^1^Department of Neurobiology, Care Sciences and Society, Center for Alzheimer Research, Division of Neurogeriatrics, Karolinska Institutet, Stockholm, Sweden; ^2^Department of Neurobiology, Care Sciences and Society, Division of Clinical Geriatrics, Karolinska Institutet, Huddinge, Sweden; ^3^Department of Neurology and Neurosurgery, McGill University, Montreal, QC, Canada; ^4^Neurogeriatric Clinic, Karolinska University Hospital, Huddinge, Sweden; ^5^University of Turku, Turku University Hospital Kiinanmyllynkatu, Turku, Finland; ^6^Translational and Clinical Research Institute, Newcastle University, Newcastle upon Tyne, United Kingdom; ^7^Department of Geriatrics, Turun Kaupunginsairaala, University Hospital of Turku, University of Turku, Turku,Finland

**Keywords:** CADASIL, GLUT4, GLUT2, VSMCs, stroke

## Abstract

Typical cerebral autosomal-dominant arteriopathy with subcortical infarcts and leukoencephalopathy (CADASIL) is caused by mutations in the human NOTCH3 gene. Cerebral autosomal-dominant arteriopathy with subcortical infarcts and leukoencephalopathy is characterized by subcortical ischemic strokes due to severe arteriopathy and fibrotic thickening of small vessels. Blood regulating vascular smooth muscle cells (VSMCs) appear as the key target in CADASIL but the pathogenic mechanisms remain unclear. With the hypothesis that brain glucose metabolism is disrupted in VSMCs in CADASIL, we investigated post-mortem tissues and VSMCs derived from CADASIL patients to explore gene expression and protein immunoreactivity of glucose transporters (GLUTs), particularly GLUT4 and GLUT2 using quantitative RT-PCR and immunohistochemical techniques. *In vitro* cell model analysis indicated that both GLUT4 and -2 gene expression levels were down-regulated in VSMCs derived from CADASIL patients, compared to controls. *In vitro* studies further indicated that the down regulation of GLUT4 coincided with impaired glucose uptake in VSMCs, which could be partially rescued by insulin treatment. Our observations on reduction in GLUTs in VSMCs are consistent with previous findings of decreased cerebral blood flow and glucose uptake in CADASIL patients. That impaired ability of glucose uptake is rescued by insulin is also consistent with previously reported lower proliferation rates of VSMCs derived from CADASIL subjects. Overall, these observations are consistent with the development of severe cerebral arteriopathy in CADASIL, in which VSMCs are replaced by widespread fibrosis.

## Introduction

Cerebral autosomal-dominant arteriopathy with subcortical infarcts and leukoencephalopathy (CADASIL) is a hereditary disease resulting from mutations of the NOTCH3 gene (Joutel et al., 1996). The initial clinical manifestation often presents as migraine with aura or minor cerebral attacks (Burkett and Dougherty, 2017) (which usually occurs during the third decade of life) and progresses to subcortical ischemic attacks, mood disturbance, cognitive decline, encephalopathy and white matter (WM) changes (Choi, 2015; Di Donato et al., 2017).

The pathological hallmarks of CADASIL are (i) a finding of granular osmiophilic material (GOM) in close proximity to vascular smooth muscle cells (VSMCs) and (ii) fibrotic thickening of small penetrating arteries. GOM can be observed under electron microscopy from skin biopsy samples, making it a suitable primary diagnostic indicator for CADASIL. Additionally, the fibrotic thickening of small vessels results in stenosis, thereby reducing blood flow and causing the ischemic attacks characteristic of CADASIL (Wang, 2018). Histopathological findings show there is severe degeneration of brain VSMCs surrounding the thickened arteries, with the cause being investigated under various hypotheses (Tikka et al., 2014). Some studies suggest that the reduced number of VSMCs is a consequence of increased apoptosis (Wang et al., 2002; Formichi et al., 2009). Other studies have reported that a reduced quantity of VSMCs may reflect a lower proliferation rate (Takahashi et al., 2010) rather than a higher apoptotic rate (Wang et al., 2002; Ihalainen et al., 2007; Viitanen et al., 2013).

In contrast to the ongoing investigations on proliferation and apoptotic rates in VSMCs (Takahashi et al., 2010), no study to-date is available that investigates the metabolic requirements and possible metabolic deficiencies in VSMCs of patients with CADASIL. Another unexplored issue concerns the lack of investigation of a possible association between glucose transport and CADASIL, particularly in VSMCs, although numerous studies have reported association between NOTCH3 and glucose transporters (GLUT) in other disease models (Santio et al., 2016; Altalhi et al., 2017; Chang et al., 2017).

Glucose is the main energy source in mammalian cells and its movement across the plasma membrane is facilitated by transporters, mainly GLUTs (Szablewski, 2017). Although several GLUTs have been identified in VSMCs, we decided to focus on GLUT2 and -4 as they have previously been implicated in other disease models (Pyla et al., 2013). GLUT4 is an insulin-dependent GLUT (Brosius et al., 1992; Cooper et al., 1993; Standley and Rose, 1994; Kahn et al., 1995; Banz et al., 1996) whereas GLUT2 is, in contrast, an insulin-independent transporter (Pyla et al., 2013). In non-stimulated cells, GLUT4 exists predominately perinuclear, but upon insulin stimulation it is translocated to the cell membrane for active transport of glucose into the cell (Park et al., 2005). In hypertension animal models, both aortic and carotid artery VSMCs show a several-fold decrease in GLUT4 gene expression, followed by reduced glucose uptake ability (Atkins et al., 2001). Interestingly, these animal models show GLUT4 insulin insensitivity with regard to VSMCs, but this phenomenon is not observed in other vessels suggesting that these VSMCs are key pressure regulators and more prone to insulin insensitivity than other VSMCs (Atkins et al., 2001). In other diseases, such as diabetes, GLUT4 seems to have been down regulated (Marcus et al., 1994), thereby indicating a lowered glucose uptake capacity in VSMCs with a possible consequence in contractibility of the blood vessels.

The most common technique for studying the role of glucose metabolism in CADASIL patients is positron emission tomography (PET) scan with 18-fluorodeoxyglucose (FDG; Tuominen et al., 2001, 2004; Tatsch et al., 2003). Early studies have implicated low cerebral blood flow as the cause of low glucose uptake in the central nervous system (Tuominen et al., 2004). It is reasonable to extrapolate from those findings that CADASIL patients would exhibit lower cerebral blood flow due to small vessel stenosis, which in turn would result in a reduced cerebral glucose metabolic rate estimated by FDG-PET (Tuominen et al., 2004; Yoon et al., 2015). In fact, a correlation has been demonstrated between low cerebral blood flow and hypo-metabolism of glucose assessed by FDG-PET (Tuominen et al., 2001). However, impaired glucose metabolism assessed by 2-deoxy-D-glucose (2DG-) or FDG-PET could also be caused by reduced total glucose transport into the brain rather than only neuronal hypometabolism *per se* since FDG-PET measures the amount of the phosphorylated tracer (and, hence, trapped glucose analogs in the brain cells) as opposed to actual cellular metabolism (Southworth et al., 2003). Moreover, other studies have confirmed that CADASIL patients have glucose hypo-metabolism, but the cause of this has not been completely elucidated (Tatsch et al., 2003).

Reduced glucose uptake could also explain both atrophy of VSMCs via either an altered proliferation or apoptosis rate (Takahashi et al., 2010; Viitanen et al., 2013; Panahi et al., 2018). We hypothesized that cerebral VSMCs have defective glucose uptake, which may be partially responsible for the previously described glucose hypometabolism assessed by PET in brains of CADASIL patients. In the present study, we used cerebral VSMCs-models derived from CADASIL patients and relevant controls and post-mortem brain tissues to investigate the expression level of key GLUTs. We focused on the insulin-insensitive GLUT2 and the insulin-sensitive GLUT4 in particular since GLUT4 has been implicated in other vascular diseases, such as diabetes-related cerebral small vessel disease (SVD; Vermeer et al., 2007; Palacio et al., 2014; Umemura et al., 2017).

## Materials and Methods

### Cell Lines and Culture

Cerebral VSMCs were established from genetically verified CADASIL patients with C133R mutation and control subjects as previously described (Panahi et al., 2018). Patient-derived cerebral arterial VSMC (VSMC^*R*133*C*^) as well as control VSMC (VSMC^*WT*^) cell lines were established from *post mortem* subarachnoidal branches of cerebral arteries (human cerebral arterial VSMC) by collagenase digestion as described previously (Gimbrone et al., 1974; Ihalainen et al., 2007; Tikka et al., 2012). Briefly, post-mortem brain samples were collected within 24 h of death and all cell cultures were planned and established immediately after obtaining the samples. Small blocks of brain tissue (1-2 cm × 1-2 cm) were cut, fragmented with razor blades and the tissues surrounding the vessels (diameter approximately 0.4–1 mm) were removed with sterile scalpels. The fragments were then transferred to tubes with ice-cold 20 mM HEPES in Dulbecco’s modified Eagle’s medium (DMEM; Life Technologies, United States), centrifuged for 5 min at 500 × *g* at RT. After discarding the supernatant, tissues were resuspended in 0.05% collagenase/dispase mixture (100 μg/mL, Roche) in 20 mM HEPES in DMEM, and incubated for 5 min at RT. The tissues were homogenized usinga10 mL pipette for every 5 min and the undigested material was removed by centrifugation at 1000 × *g* for 10 min at RT. The pellets were washed with 10 mL of PBS (with calcium and magnesium) four times following resuspension in 10 mL complete culture medium containing 10% FBS 2 mM L-glutamine, 100 U/mL penicillin and 100 μg/mL streptomycin at RT. Approximately, 1.5 mL aliquots of the free vessels in solution were transferred into sterile 12-well cell culture plates and incubated at 37°C and 5% CO_2_ in a humidified environment. The media were changed every 2^*nd*^ day and once the vessels had settled down at the bottom of the wells (∼3 days) observing out-crawling cells from the tissues and their confluency, the smooth muscle cells were harvested using 0.25% trypsin for further culturing into 35-mm cell culture dishes (1:1.23 surface ratio) (Gauthier et al., 2012). For partial immortalization, the cells were infected with a human papilloma virus construct E6/E7 at early passage (p1 to p3). The infection was verified by culturing the cells in the presence of G418 (Invitrogen, Auckland, NZ, United States) (400 μg/mL) for a 10-day period. Primary cells (passage 1) were confirmed to be VSMC using the marker α-smooth muscle actin (α-SMA). After the viral infection (passage 2–5), all VSMC lines were screened negative for mycoplasma using the VenorGeM mycoplasma detection kit (Minerva Biolabs GmbH, Berlin, Germany) and with DAPI staining.

Vascular smooth muscle cells (CADASIL and control) were routinely cultured in DMEM]/F-12, GlutaMAX medium (Life Technologies, United States) supplemented with 10% heat inactivated fetal bovine serum (FBS), 1% Penicillin-Streptomycin and 1% L-glutamine. The cell lines were kept in an incubator with 5% CO_2_ at 37°C. Passages of cells were matched for each experiment and were between 18 and 28. Umbilical artery smooth muscle cells (UASMC, Lonza) were cultured in smooth muscle cell medium BulletKit according to the manufacturer’s instructions or cultured in M 231 medium with smooth muscle growth supplement (Life Technologies) (Panahi et al., 2018).

### Real Time Quantitative PCR

Vascular smooth muscle cells (CADASIL and control) were grown overnight in a six-well plate chamber with confluence of 100,000 cells. The following day, the cells were lysed with RIPA buffer (Thermo Fisher Scientific, United States) and the quality of RNA was determined with RIN (RNA Integrity Number) of 10. cDNA was prepared using Taqman gene expression master mix (Applied Biosystem) and SuperScript VILO cDNA Synthesis kit (Thermo Fisher Scientific, United States) according to the manufacturer’s protocol. Quantitative (q)RT-PCR was performed using costume format TaqMan fast plate (Applied Biosystems; No. 4427562, Rev C)^1^. The gene designations for control and *GLUTs* were: *HPRT1*-Hs99999909_m1 (endogenous control), *GAPDH*-Hs99999905_m1 (endogenous control), 18S-Hs99999901_s1, *SLC2A2*-Hs01096908_m1; *SLC2A3*-Hs00359840_m1, and *SLC2A4*-Hs00168966_m1. All probes were used in duplicates with 30 ng of cDNA. Quantitative RT-PCR was performed on a 7500 Fast Real-Time PCR System (Life Technologies, United States). The expressions of genes were normalized to internal control HPRT gene and analysis was used comparing the normalized value of control cell line to the normalized values of CADASIL cell line. All the quantitative data were from three independent biological replicates for each experiment and the control value was normalized to 1.

### Immunofluorescent Staining of GLUT4 in VSMC Cells

For VSMCs staining, 3.0 × 10^4^ cells were seeded onto 8-well chamber slides (Lab-Tek) for 48 h. The cells were grown until 80% confluency, harvested, washed twice with PBS and fixed with paraformaldehyde (PFA; 4% v/v, Sigma-Aldrich) for 10 min. Following blocking with the Bovine Serum Albumin (BSA; 1% w/v) for 30 min, the cells were permeabilized with Triton X-100 (0.1% v/v, Sigma-Aldrich) for 30 min at RT. Later, cells were incubated with goat anti-GLUT4 (C-20m Santa Cruz) primary antibody overnight at 4°C. Subsequently, the cells were washed and incubated with conjugated secondary Alexa Fluor 488 antibody for 1 h at RT. The slides were mounted with DAPI. Vascular smooth muscle cells were examined using a laser scanning confocal microscope (LSM 510 META, ZEISS).

### Immunoblotting of VSMCs

Vascular smooth muscle cells (CADASIL and control) grown to 80% confluence were harvested by scraping from the plate after washing twice with PBS. Cells were collected by 5 min centrifugation at 300 *g*, and lysed in lysis buffer (0.65% NP40, 10 mM Tris pH 8.0, 1 mM EDTA, 150 mM NaCl) containing protease inhibitor (100×, ProteaseArrest G-Biosciences). The total protein content was measured using the Pierce bicinchoninic acid (BCA) protein assay kit (Thermo Fisher Scientific). Laemmli sample buffer (2× LDS, Sigma-Aldrich) was added to the samples. 2× LDS sample buffer (Invitrogen) was used for SDS-PAGE and immunoblotting as previously described (Behbahani et al., 2010). For GLUT4, a rabbit polyclonal antibody (H-43: sc-7903, Santa Cruz Biotechnology), and rabbit polyclonal Cpt1C (carnitine palmitoyltransferase I) antibody (Supplementary Figure S1) was purchased from abcam (ab87498, United States).

### Functional Glucose Uptake Assay Using NBDG-Flow Cytometry

First, the cells were incubated and grown in DMEM (Life Technologies, United States) without the additional FBS and glucose for 24 h to a monolayer confluence. Later, the medium was removed and cells were washed 3× with PBS Krebs ringer phosphate buffer with 50 mM glucose and 200 μM 2-NBDG (2-(*N*-(7-Nitrobenz-2-oxa-1,3-diazol-4-yl)Amino)-2-Deoxyglucose) (Thermo Fisher Scientific, United States) was added to the cell layer. Cells were incubated with and without 50 mM insulin for 30 min. The cells were harvested and the 2-NBDG uptake was measured by flow cytometry using a FACSCalibur^TM^ Cytometry (BD Biosciences, United States). Approximately, 10,000 events were collected for each analysis. Data from the experiments were analyzed using the CellQuest software (BD Biosciences, United States).

### Human Brain Tissues

Table 1 provides demographic details and diagnoses in the subjects used in immunohistochemical studies (Craggs et al., 2014). The mean age of CADASIL subjects were not different from mean ages of controls. Available case notes and radiological reports indicated that CADASIL subjects showed extensive WM changes consistent with SVD of the brain and met the minimum criteria for cognitive impairment (Allan et al., 2011; Skrobot et al., 2018). Majority of the CADASIL subjects had no known vascular disease risk (Craggs et al., 2014). However, one patient each had exhibited cardiac arrythmias, obesity and >10 year smoking history. The diagnosis of CADASIL was confirmed by the presence of *NOTCH3* gene mutations or the presence of GOM in arteries within skin biopsies (Yamamoto et al., 2009). None of the controls had neurological or pathological evidence for cerebrovascular disease or neurodegenerative disorder.

**TABLE 1:** Demographic details of CADASIL subjects and controls.

Group (*n*)^#^	Age (year) (Range)	Gender distribution	*NOTCH3* Mutation Type (no)	Duration (year)	Notable clinical features and risk factors
CADASIL* (14)	59.0 ± 8.0 (33–74)	9M/5F	Arg133Cys (6); Arg141Cys (2); Arg153Cys (2); Arg169Cys (2); Arg558Cys (1); Arg985Cys (1)	6–28	Majority of cases did not have preexisting vascular risk factors. Cardiac arrhythmias (1); Brief history of gout (1); obesity (1); prostate tumor (1); smoking history (3)
Controls (13)	65.7 ± 8.1 (49–78)	5M/8F	–		No significant cerebrovascular or neurodegenerative disorder. No pathological diagnosis

*Cases and controls used for the various experiments. ^∗^The MMSE scores for the patients ranged from 12 to 24 (not available for all cases). Controls, mean age was not significantly different from that of CADASIL group (*p* > 0.05). ^#^There are three samples each from the Swedish Brain Bank.*

For immunohistochemical analyses, the brain tissues from CADASIL subjects (frontal and occipital lobes) and aged-matched controls were from the Newcastle Brain Tissue Resource (NBTR), Newcastle University, Campus for Ageing and Vitality, and the Swedish Brain Bank (Stockholm, KI). All studies involving human subjects have been approved by the local research ethics committee of the Newcastle; the NBTR committee, and the Regional Ethical Review Board in Stockholm or the Research Ethics Committee of the South Huddinge University Hospital.

### Immunofluorescence Staining and Confocal Microscopy

Immunofluorescence staining was performed on 5 μm sections of formalin-fixed paraffin-embedded (FFPE) frontal cortex of post-mortem brains. The sections were de-paraffinzed and hydrated through xylene and graded alcohol series. The sections were autoclaved with antigen retrieval buffer (DV2004, DIVA Decloaker, Biocare Medical) for 30 min at 110°C (Decloaking Chamber NxGen, Biocare Medical). After the temperature decreased to room temperature (RT), sections were washed with water for 5 min and then in Tris-Buffered Saline (TBS) + 0.05% Tween 20 (TBS-T) (91414, Sigma-Aldrich). The sections were blocked with Background Punisher (BP974, Biocare Medical) for 10 min at RT followed by washing and incubation with primary antibodies; α-SMA antibody (Abcam, SMA, 1:500, Clone 1A4, Abcam, United States), as an indicator for VSMC, and GLUT4 (ab48547, 1:500, Abcam, United States) and GLUT2 antibodies (ab54460, 1:500, Abcam, United States), in TBS-T (overnight 4°C, humid chamber).

After washing in TBS-T, sections were incubated (1 h at RT) with appropriate secondary antibodies [anti-mouse and anti-rabbit IgG (H + L)] conjugated to Alexa Fluor 546 or Alexa Fluor 488 (Invitrogen) at a concentration of 1:500 in TBS-T. To reduce auto-fluorescence, the slides were further washed in TBS-T (3 × 10 min) and incubated with or without Sudan Black B (199664, Sigma-Aldrich), 5 min at RT. Sections were then washed in TBS-T and were mounted with DAPI Vectashield Hard Set (H-1200, Vector Laboratories) and the slides were stored at 4°C. Sections from controls and patients were also incubated without primary antibody and used as negative control. Quantification of cerebral vessels was undertaken essentially as described previously (Craggs et al., 2015). We focused on vessels of size 50-300 μm in diameter consisting largely of arterioles. The appropriate gray and while matter boundaries were delineated by the DAPI background staining within tissue sections.

### Imaging Analysis

Sections were examined using a laser scanning confocal microscope (LSM 510 META, ZEISS, or core Facility Bionut Microscopy), and images were acquired using the same settings (laser intensity, detector gain and amplifier offset). Also, the tissues fluorescence staining was recorded sequentially in separate channels with Plan-Apochromate 20× (NA, 0.8), 40× (NA, 1.2) oil and 100× (NA, 1.45) oil objectives. Image processing was performed with the included ZEN software. Cell quantifications were performed with Neurolucida. Fluorescence intensity was measured with the ImageJ 1.383 software (NIH, MA, United States).

### Statistical Analyses

Statistical comparison of values between groups was measured by one-way ANOVA followed by Bonferroni’s *post hoc* test. Student *t*-test was used for two-group comparisons. *p*-values < 0.05 were considered significant. The results are representative of three independent biological replicates expressed as mean ± SEM. All univariate and multivariate analyses were performed using *IBM SPSS Statistics* software (version 23.0). A two-tailed *p*-value of <0.05 was considered as the threshold for statistically significant differences or associations in all analyses.

## Results

### GLUT4 Is Down-Regulated in CADASIL VSMCs

To shed light on the mechanism of previously observed lower FDG-PET value in the CADASIL subjects (Tuominen et al., 2004), we investigated gene expression levels of GLUT isoforms [*SLC2A2* (GLUT2), *SLC2A3* (GLUT3), and *SLC2A4* (GLUT4)] in VSMC models of CADASIL and control subjects.

**FIGURE 1:**
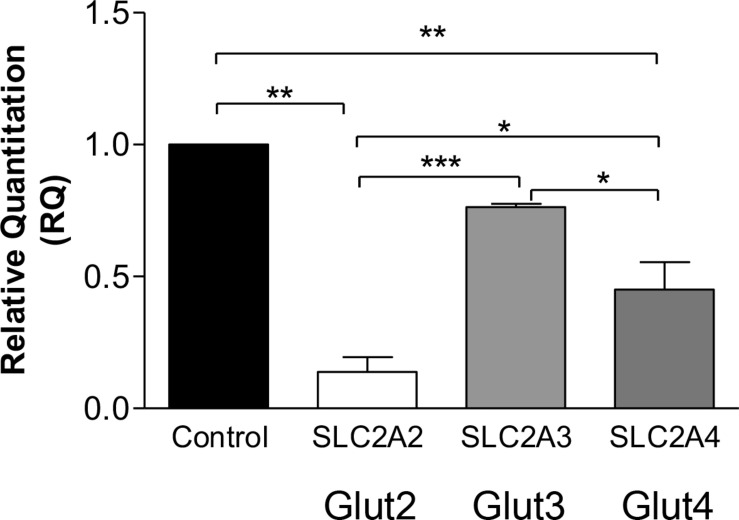
Glucose transporters gene expression in CADASIL compared to control VSMCs. qRT-PCR analysis of GLUT2 (*SLC2A2*), GLUT3 (*SLC2A3*), and GLUT4 (*SLC2A4*), genes in CADASIL and control VSMCs. The expression of GLUT2, GLUT3, and GLUT4 genes were normalized to the endogenous control gene; HPRT1, and the RQ (Relative Quantitation) was calculated using control VSMC normalized to 1. *p < 0.05, **p < 0.01 and ***p < 0.001. The results are representative of three independent replicates (*n* = 3). One-way ANOVA followed by Bonferroni’s *post hoc* test was used for statistical analysis.

Quantitative RT-PCR revealed significantly decreased *SLC2A2* and *SLC2A4* gene expressions in CADASIL VSMCs compared to controls (*p* < 0.001, and *p* < 0.01, respectively) (Figure 1). *SLC2A3* gene expression tended to be lower but was only different from *SLC2A2* and *SLC2A4.* Since the insulin-dependent transporter GLUT4 was previously assessed in hypertension models and in diabetes as a model of small vessel disease, we focused on this specific transporter for the gene product studies in relation to the insulin-independent GLUT2. In view of the findings on *SLC2A3* gene expression we did not perform further protein studies on GLUT3.

**FIGURE 2:**
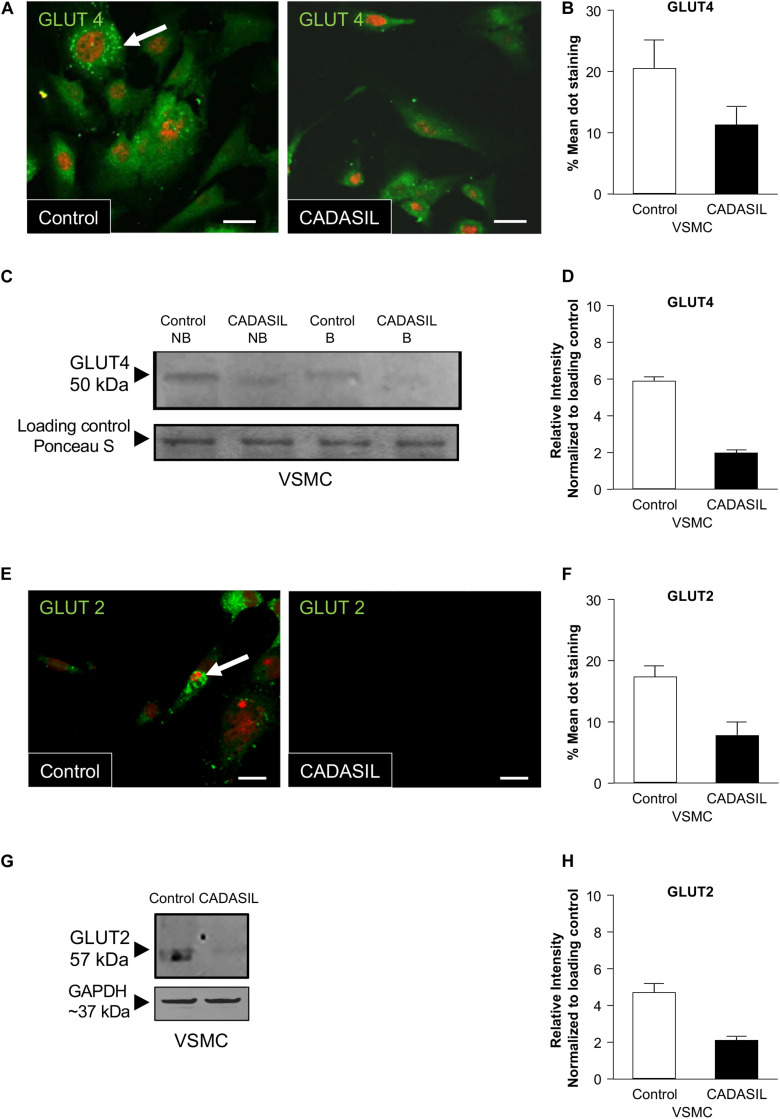
GLUT4 expression in CADASIL compared to control VSMCs. PFA-fixed VSMCs were immunostained with goat anti-GLUT4 and GLUT2 antibodies, visualized using confocal microscopy and assessed for expression of the GLUTs. **(A,E)** Images of VSMCs stained for GLUT4 and GLUT 2 using an Alexa Fluor-conjugated 488 secondary antibody. GLUT dot-staining is shown in green (arrows) and Nuclei are stained with DAPI (here showed in fluoresces red). Representative images were taken from one experiment (*n* = 3), Scale bar, 10 μm. **(B,F)** Mean values of GLUT4 and GLUT2 dot-staining in control and CADASIL VSMCs. Each data point represents mean of intensely fluorescent GLUT-positive dots per cell in 12 cells from 3 independent experiments (*n* = 3) (*p < 0.05). **(C,G)** Immunoblotting analyses, non-boiled (NB) and boiled samples (B) were conducted to assess the expression of GLUT4 in VSMCs. The samples were visualized using the Odyssey CLx Imager. 15 μg of protein was loaded into the gel and ponceau S staining was used as loading control. The intensity of the GLUT4 and GLUT2 band was lower than in both CADASIL boiled and non-boiled samples as compared to control VSMC lysates **(D,H)** Densitometry data from CADASIL and control protein bands normalized to Ponceau S total protein stain showed lower expression of GLUT4 and GLUT2 in CADASIL VSMCs than in controls. The immunoblot is representative of one experiment.

Using confocal microscopy analysis, we also observed reduced GLUT4 and GLUT2 expressions in CADASIL VSMCs. Quantification of GLUT4 and GLUT2 distribution in these cells showed significant lower expression of GLUTs in CADASIL VSMCs compared to controls (Figures 2A,B,E,F). Furthermore, immunoblotting analysis showed reduced GLUT4 protein levels in CADASIL VSMCs compared to control (Figures 2C,D,G,H). These data together suggest that the glucose uptake receptors are impaired in these arterial cells.

### Glucose Uptake Is Not Affected by Insulin in CADASIL VSMCs

Low GLUT expression in CADASIL VSMCs suggests that these cells have potentially lower glucose uptake ability. VSMCs derived from CADASIL also exhibited a lower proliferation rate compared to VSMCs from control (Panahi et al., 2018). Therefore, we hypothesized that lower proliferation rate might be due to the lower glucose uptake ability of VSMCs in CADASIL.

**FIGURE 3:**
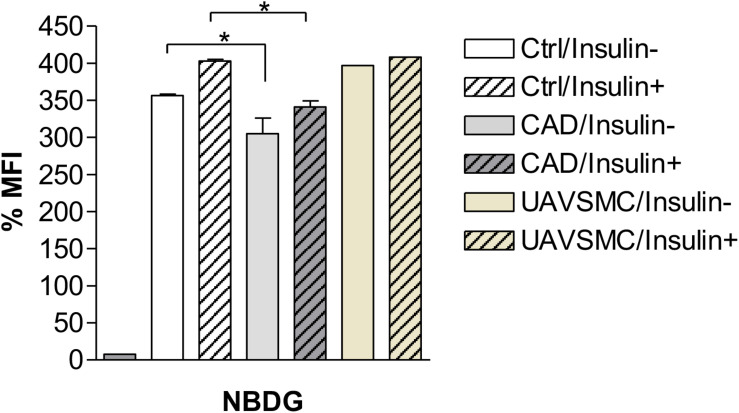
Effects of Insulin on Glucose uptake in CADASIL VSMCs. The VSMCs (CADASIL, control and UAVSMCs) were cultured and 200 μM 2-NBDG; a fluorescent indicator for monitoring glucose uptake, was added to the cells (M&M), incubated with and without 50 mM insulin. The 2-NBDG uptake was measured by flow cytometry. Flow cytometry analysis showed a reduced glucose uptake before addition of insulin in CADASIL VSMCs (*p < 0.05), however, lower glucose uptake was not affected by insulin in CADASIL VSMCs. Ctrl: control VSMC, CAD: CADASIL, MFI: mean fluorescence intensity, UAVSMC: Umbilical artery smooth muscle cells.

An experimental procedure was set up to measure the uptake of glucose in both CADASIL and control VSMCs. Validation of glucose uptake was carried out using 2-NDBG, a fluorescent indicator for monitoring glucose uptake into living cells, which could be assessed by flow cytometry. Flow cytometry analyses showed that CADASIL VSMCs had a significantly lower glucose uptake capability (*p* < 0.05), most likely reflecting the lower gene expression of GLUT in CADASIL VSMCs (Figure 3). These analyses indicated that the reduced gene expression was in line with an overall functional effect.

To further confirm the above findings, we assessed the extent to which insulin could rescue the deficit in glucose uptake in the cellular model of CADASIL. The results indicated that insulin rescued glucose uptake by ∼10% in both CADASIL and control VSMCs. Since GLUT4 is an insulin-sensitive transporter, the findings suggest that GLUT4 is functional in CADASIL VSMCs and, hence, the observed differences are mainly related to an altered GLUT-4 gene expression in CADASIL VSMCs (Figure 3). Considering that gene expression analyses indicated that reduced gene expression of GLUTs occurs in the following order GLUT2 > GLUT4 > GLUT3 in CADASIL VSMCs (see Figure 1) and that GLUT2 is an insulin-independent transporter, the functional result here, further suggested that overall altered glucose uptake in CADASIL VSMCs is caused by both insulin-dependent and insulin-independent transport mechanisms. We also found that carnitine palmitoyltransferase I (Cpt1), specifically the Cpt1c isoform, highly expressed in brain (Schreurs et al., 2010), did not differ between the control VSMCs and CADASIL VSMCs (Supplementary Figure S1). This indicated that the reduced glucose uptake in VSMCs was not associated with changes in beta-oxidation of fatty acids and insulin sensitivity in the VSMCs.

### GLUT4 and GLUT2 Expression Level in Brain Tissues From CADASIL Subjects

**FIGURE 4:**
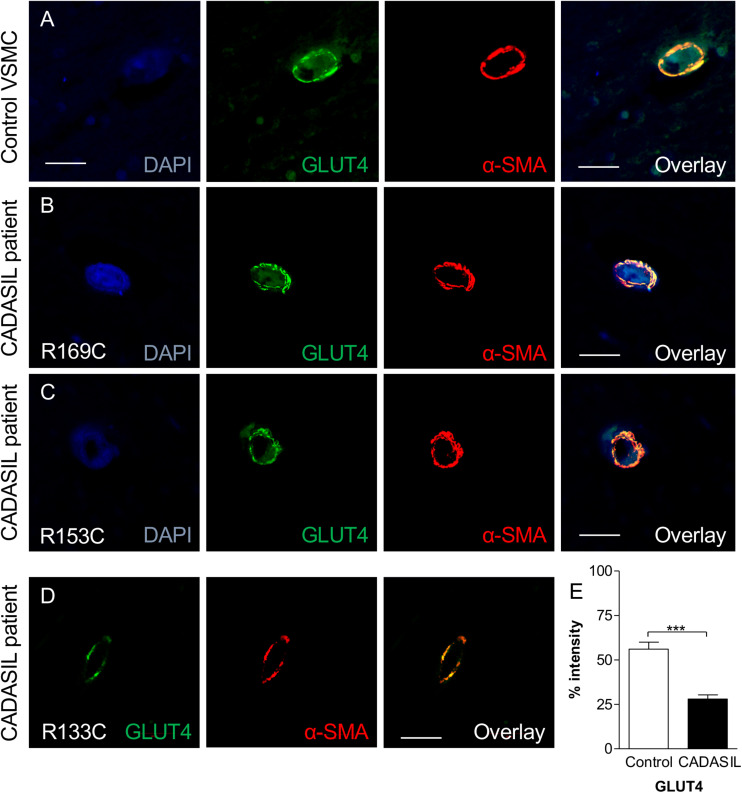
GLUT4 immunoreactivity in VSMC in frontal white matter in CADASIL. **(A)** Immunofluorescent labeling with GLUT4 (green) and α-SMA (red) antibodies (Nuclear, DAPI, blue) is shown. A strong GLUT4-immunostaining in control within the cortex was observed. GLUT4 staining in CADASIL patient carrying **(B)** p.Arg169Cys mutation, **(C)** p.Arg153Cys, and **(D)** p.ArgR133Cys mutation is shown. Magnification bar = 20 μm in panels **(A,B,D)**, and 50 μm in panels **(C)**. **(E)** Quantification of GLUT4 expression demonstrated significantly lower GLUT4 expression in CADASIL brain tissues as compared to controls (***p < 0.001). CADASIL, cerebral autosomal dominant arteriopathy with subcortical infarcts and leukoencephalopathy. α-SMA: anti-smooth muscle alpha-actin.

Our observations were based on cellular experiments derived from patients with the *NOTCH3* C133R mutation. However, to validate our findings, and to test if there was any association between GLUTs and other mutations, we assessed the expression level of GLUT2 and GLUT4 in post-mortem brain tissues from CADASIL and controls using immunohistochemical techniques and analyzed the results using confocal microscopy. Confocal analysis of multiple brain tissues revealed increased GLUT4 immunoreactivity in control samples compared to CADASIL samples (Figure 4). Furthermore, an association of GLUT4 expression with different *NOTCH3* mutations was observed in CADASIL subjects compared to controls (Figures 4A–D). We also found that there were no clear differences in GLUT expression in the 3 CADASIL subjects who exhibited previous vascular disease risk compared to those who did not. Preliminary observations indicated that there was also a correlation between vessel diameter and GLUT4 expression (data not shown), but further investigation is needed to confirm this finding.

**FIGURE 5:**
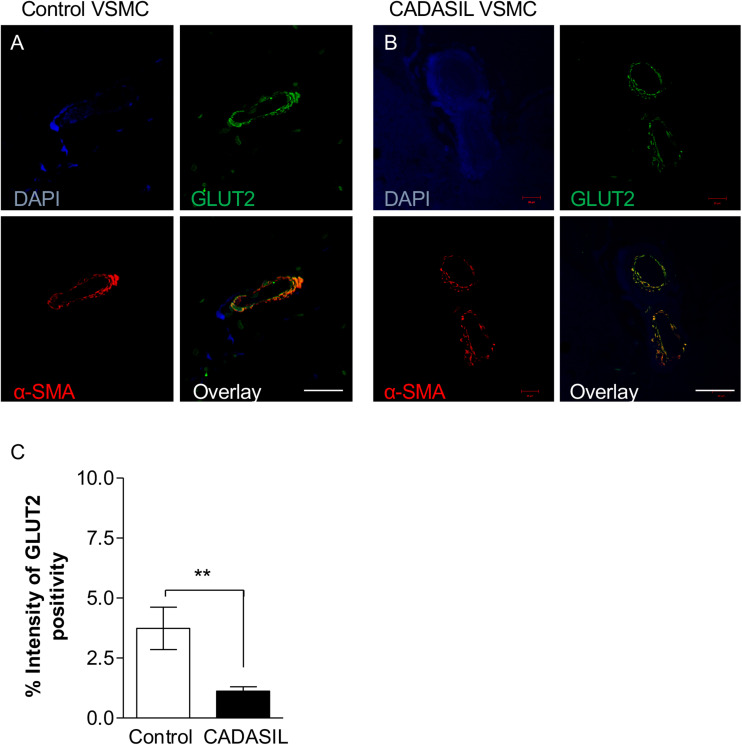
GLUT2 immunoreactivity in cerebral microvessels in CADASIL. Immunofluorescent labeling of GLUT2 (green), smooth muscle α-actin (SMA; red) and arteries and arterioles in the frontal white matter, counterstained with DAPI **(A,B)**. Sections from panel **(A)** a 94-year-old non-demented female control and **(B)** 68-year-old female CADASIL case with p.Arg133Cys mutation. Severe capillary degeneration was observed in CADASIL **(B)**, with low GLUT2 expression observed in VSMCs compared to control subject. There was minimal SMA immunoreactivity in capillaries in CADASIL. Scale bar = 20 μm in panels **(A,B)**. **(C)** Quantification of GLUT2 expression demonstrated significantly lower GLUT2 expression in CADASIL brain tissues as compared to controls (**p < 0.01). CADASIL: cerebral autosomal dominant arteriopathy with subcortical infarcts and leukoencephalopathy.

We observed that the smaller the vessel in CADASIL, the less GLUT4 is expressed compared to controls. Overall, quantitative analysis demonstrated lower GLUT4 protein expression in small vessels in CADASIL compared to control subjects (*p* < 0.001, Figure 4E). Similarly, quantitative immunostaining of GLUT2 in small vessels (Figures 5A,B) indicated significantly lower levels of GLUT2 in CADASIL compared to controls (*p* < 0.01, Figure 5C). In accord with our previous work (Craggs et al., 2014), we concentrated on the WM but we found that invariably arteriolosclerotic vessels in the gray matter also exhibited GLUT reduction (data not shown).

Nonetheless, based on the dual immunofluorescence labeling of GLUT2 and GLUT4 in relation to α-SMA (Figures 4, 5), the altered levels of GLUT2 and GLUT4 may reflect an altered gene expression in GLUTs in smooth muscle component of the small blood vessels. This is because the dual immuno-staining analysis indicated co-localized staining between GLUT2/4 positive cells and α-SMA. Thus, these findings were consistent with the relative changes observed in the GLUT2 and GLUT4 gene expression analyses in the cellular models of CADASIL versus control (compare Figure 1 and Figures 4E, 5C). Remarkably, the relative changes in GLUT2 immunoreactivity were lower than the corresponding GLUT4 immunoreactivity in these small vessels. There was ∼ 67% decrease in GLUT2 compared to ∼50% inGLUT4 relative to controls (cf. Figures 4E, 5C).

## Discussion

We showed here for the first time that the gene and protein expression of the main GLUTs, GLUT2 and GLUT4 are altered in both cellular models as well as post-mortem brain of CADASIL patients compared to controls. It is difficult to set specific thresholds on what degree of reduction in GLUTs would alter physiological conditions. However, we were careful to compare like VSMCs from controls and patients that were isolated, maintained and cultured in parallel in a similar manner. Irrespective of how the previously reported reduction in glucose metabolism using FDG-PET analyses (Southworth et al., 2003; Tatsch et al., 2003; Tuominen et al., 2004) is interpreted or whether there is true reduction in cellular metabolism or in glucose uptake, we found GLUTs were reduced in the CADASIL VSMCs models and brain microvessels or arterioles of CADASIL patients in the order: GLUT2 > GLUT4 > GLUT3. These findings suggest the possibility that to some degree the profoundly reduced FDG-PET coupled with low cerebral blood flow (CBF) values in CADASIL patients reflect reduced glucose uptake rather than only cellular hypo-metabolism. This suggestion is not inconsistent with the previous findings, albeit in normal aging and Alzheimer’s disease subjects that deficits in CBF and cerebral metabolic rate for glucose determined by FDG-PET as in CADASIL could more significantly reflect degradation of the *K_1_^∗^* parameter (transport of glucose) rather than the *k_3_^∗^* (phosphorylation) step (Jagust et al., 1991; Fukuyama et al., 1994).

GLUT4 has also previously been shown to exhibit an altered expression level in arterial myocytes in different disease models (Marcus et al., 1994; Zhang et al., 1999; Atkins et al., 2001). Here, we showed that CADASIL VSMCs expressed lower levels of GLUT4 and GLUT2 both in post-mortem brain tissues and VSMCs derived from CADASIL patients. It is unclear whether NOTCH3 may regulate GLUT4 and GLUT2 directly or via a non-covalent binding mechanism. In preliminary experiments, we were unable to detect co-immunoprecipitates of purified NOTCH3 and either GLUT4 or GLUT2. However, NOTCH3 could alter their expression via another protein that is currently elusive or the expression is related to the reduced glucose metabolism during lower proliferation rates of VSMCs with *NOTCH3* mutants (Takahashi et al., 2010).

It is plausible that reductions in arteriolar GLUT4 and other GLUTs including capillary GLUT1 could impact on WM integrity. Our previous observations showed there is severe WM arteriopathy and capillary degeneration in CADASIL (Craggs et al., 2014; Hase et al., 2019). Thus, if the vascular entry routes for glucose are impaired or that arterial muscle cells exhibit reduced GLUTs arteriosclerosis may ensue to affect perfusion and flow affecting the WM. This is consistent with previous observations of reduced WM CBF (Tuominen et al., 2004) and frontal WM pathology and axonal disconnectivity in CADASIL (Craggs et al., 2014). However, NOTCH3 mutations could also have a direct effect on WM cells not necessarily involving the microvasculature (Rajani et al., 2019).

Among the known GLUTs, GLUT4 is the main insulin-dependent transporter, which makes it a suitable candidate for developing a potential medical intervention. Nonetheless, our results indicated that GLUT2 had the most reduced levels in both cellular models and the brain VSMCs of the patients. Indeed, our functional glucose uptake experiment indicated that a significant proportion of glucose uptake was through non-insulin dependent transporters. A previous work based on CADASIL endothelial cells (ECs) showed abnormalities in GLUTs in ECs (Mann et al., 2003). GLUT1 is the major glucose transporter in ECs, and its expression level is decreased in CADASIL as shown in brain biopsy studies (Giwa et al., 2012; Craggs et al., 2013; Craggs et al., 2015). Since GLUTs are expressed in VSMC of systemic organs, and there are few studies that indicate that the VSMC of other organs are bear GOM deposits in CADASIL subjects (Lorenzi et al., 2017) and that endothelium-dependent vasodilation is impaired in forearm resistance arteries (Stenborg et al., 2007) it is not unlikely that GLUT4 and -2 are affected in other organs of CADASIL patients. We were not able to analyze GLUTs in other organs VSMC due the unavailability of systemic tissues. Since CADASIL is largely characterized as a cerebral small vessel disease with consequent brain pathology and dysfunction we focused on the brain. Furthermore, the brain microvasculature is more of interest because it is endowed with the blood-brain barrier unlike systemic the vessels. Further research is needed to clarify the disruption of glucose metabolism in VSMCs derived from other organs and ascertain whether this phenomenon is only seen in VSMCs derived from brain vessels.

We found that the expression of GLUT varied based on blood vessel size, which is in accord with the variable densities of VSMCs in different size vessels (Craggs et al., 2015). Another recent report indicates that GLUT4 may act as one of the regulators of the tonic capacity of VSMCs (Atkins et al., 2015), suggesting that in CADASIL the blood flow restriction might be, in part, due to reduced GLUT4 (and GLUT2).

Cerebral autosomal-dominant arteriopathy with subcortical infarcts and leukoencephalopathy causes degeneration in the walls of small vessels of the brain leading to lacunar infarcts and leukoencephalopathy. Like in other diseases, vascular risk factors such as hypertension and diabetes, and lifestyle modifications including obesity and smoking can also exacerbate disease progression. This may create chronic hypoxia altering GLUT4 expression and impair proliferation of VSMCs (Zhang et al., 1999; Chou et al., 2004; Takahashi et al., 2010; Szablewski, 2017; Zhao et al., 2017). The same factors might also affect expression of GLUT2, as evident in the current study. Acknowledging these factors play a role in GLUT4 and GLUT2 expressions, we attempted to normalize our data. However, given that in this study we used CADASIL brain tissue collected post-mortem, it is impossible for us to definitively control for such variables. We gathered tissue sections from different Brain Bank sources and some of these tissues are from different individuals that have the same mutation on the *NOTCH3* gene.

Another limitation in this study is that the *in vitro* data was based on a few available cerebral VSMCs cell lines models. However, we should emphasize the difficulties and limitations of preparing cerebral VSMCs derived from CADASIL patients. It was too small to address the effect of various mutations known to cause this disease. Multi-center studies with a larger number of CADASIL patients are required to address such effects.

Whether *NOTCH3* directly affects GLUT4 expression or there are other mechanisms at play remains to be clarified. Using insulin, we were able to increase glucose uptake in CADASIL VSMCs; however, this did not restore glucose uptake to the control level. These data together with lower GLUT2 expression in CADASIL subjects suggest that *NOTCH3* mutations affect the spectrum of GLUTs. Animal models would be of interest in following up the findings in this study. Since all of our experimental material is obtained from human subjects with varying genetic backgrounds, it would be most interesting to study the same phenotype using animals with the same genetic background. It would also be noteworthy to study glucose metabolism in VSMCs in animals that have both copies of *NOTCH3* mutated, considering that in our experiments the mutations are heterozygous.

## Conclusion

Our observations suggest both the insulin independent and dependent GLUT2 and GLUT4, respectively are impaired in VSMCs and WM arteries of CADASIL subjects. These findings are consistent with decreased cerebral blood flow and glucose uptake demonstrated by FDG-PET in CADASIL patients. The impaired ability of glucose uptake being rescued by insulin is further consistent with lower proliferation rates of VSMCs in CADASIL subjects. Our findings are consistent with the development of severe arteriopathy in CADASIL, in which VSMCs are replaced by widespread fibrosis.

## Data Availability Statement

All datasets presented in this study are included in the article/Supplementary Material.

## Ethics Statement

The studies involving human participants were reviewed and approved by LEC of the Newcastle Health Trust, Newcastle Brain Resource Centre the NBTR committee, and the Regional Ethical Review Board in Stockholm or the Research Ethics Committee of the South Huddinge University Hospital. The patients/participants provided their written informed consent to participate in this study.

## Author Contributions

MP, PR, and HB conceived and designed the experiments. MP, DA, NB, and PR performed the experiments. MP, PR, DA, S-MF, JR, and HB analyzed the data. MP, PR, BW, AC-M, MV, YH, RK, TD-S, and HB wrote and edited drafts of the manuscript. All authors contributed to the article and approved the submitted version.

## Conflict of Interest

The authors declare that the research was conducted in the absence of any commercial or financial relationships that could be construed as a potential conflict of interest.

## Footnotes

1 https://www.thermofisher.com/order/taqman-files

## Supplementary Material

FIGURE S1 Immunoblotting analyses were conducted to assess the expression of Cpt1c in VSMCs. The samples were visualized using the Odyssey CLx Imager. Fifteen μg of protein was loaded into the gel and GAPDH was used as loading control. Band intensity of Cpt1c immunoreactivity was similar between CADASIL and control VSMC lysates. Abbreviation: CAD: CADASIL VSMC, Crtl: Control VSMC, GAPDH, Glyceraldehyde-3-phosphate dehydrogenase.
