# Supplementary figures and images for: Insulin-Independent and Dependent Glucose Transporters in Brain Mural Cells in CADASIL
Source: Front Genet. 2020 Sep 15;11:1022. doi: 10.3389/fgene.2020.01022 (PMC7522350; doi:10.3389/fgene.2020.01022)

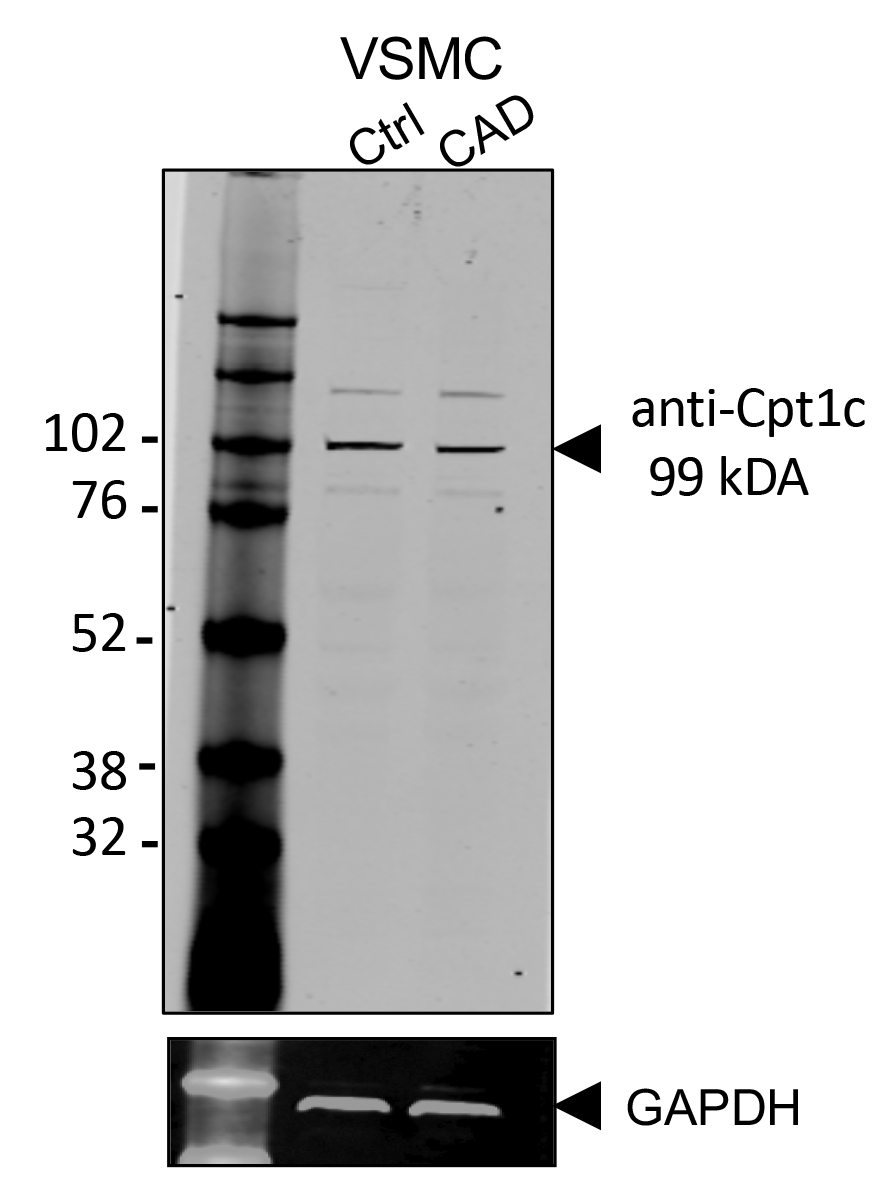

Supplement: FIGURE S1 — Immunoblotting analyses were conducted to assess the expression of Cpt1c in VSMCs. The samples were visualized using the Odyssey CLx Imager. Fifteen μg of protein was loaded into the gel and GAPDH was used as loading control. Band intensity of Cpt1c immunoreactivity was similar between CADASIL and control VSMC lysates. Abbreviation: CAD: CADASIL VSMC, Crtl: Control VSMC, GAPDH, Glyceraldehyde-3-phosphate dehydrogenase. [file Image_1.TIF]
